# Nanocapsules with Polyelectrolyte Shell as a Platform for 1,25-dihydroxyvitamin D3 Neuroprotection: Study in Organotypic Hippocampal Slices

**DOI:** 10.1007/s12640-016-9652-2

**Published:** 2016-07-15

**Authors:** Joanna Ślusarczyk, Marek Piotrowski, Krzysztof Szczepanowicz, Magdalena Regulska, Monika Leśkiewicz, Piotr Warszyński, Bogusława Budziszewska, Władysław Lasoń, Agnieszka Basta-Kaim

**Affiliations:** 1 Department of Experimental Neuroendocrinology, Institute of Pharmacology, Polish Academy of Sciences, 12 Smętna St, 31-343 Krakow, Poland; 2 Jerzy Haber Institute of Catalysis and Surface Chemistry, Polish Academy of Sciences, 8 Niezapominajek St, 30-239 Krakow, Poland

**Keywords:** Nanocapsules, 1,25-dihydroxyvitamin D3, Encapsulation, Polyelectrolytes, Hippocampal organotypic cultures, Neuroprotection, Neurodegeneration, Nitric oxide

## Abstract

Calcitriol (1,25-dihydroxyvitamin D3), an active metabolite of vitamin D3, besides the role in calcium and phosphorus metabolism, plays a role in maintaining the functions of the brain. Active forms of vitamin D3 stimulate neurotrophic factors’ expression, regulate brain immune processes, and prevent neuronal damage. Therefore, a potential utility of vitamin D3 in a therapy of neurodegenerative disorders should be taken into account. On the other hand, systemic vitamin D3 treatment carries the risk of undesirable effects, e.g., hypercalcemia. Thus, 1,25-dihydroxyvitamin D3 targeting delivery by nanoparticles would be a tremendous advancement in treatment of brain disorders. Calcitriol was enclosed in emulsion-templated nanocapsules with different polymeric shells: PLL (Poly(l-lysine hydrobromide)), PLL/PGA (/Poly(l-glutamic acid)), and PLL/PGA-g-PEG (Poly(l-glutamic acid) grafted with polyethylene glycol). The average size of all synthesized nanocapsules ranged from −80 to −100 nm. Biocompatibilities of synthesized nanocarriers were examined in hippocampal organotypic cultures in basal conditions and after treatment with lipopolysaccharide (LPS) using various biochemical tests. We demonstrated that nanocapsules coated with PLL were toxic, while PLL/PGA- and PLL/PGA-g-PEG-covered ones were nontoxic and used for further experiments. Our study demonstrated that in LPS-treated hippocampal slices, both types of loaded nanoparticles have protective ability. Our findings underlined that the neuroprotective action of vitamin D3 in both free and nanoparticle forms seems to be related to the suppression of LPS-induced nitric oxide release.

## Introduction

Despite many years of research, the neurodegenerative diseases are still medical, social, and economical problems especially in view of increasing life expectancy. Furthermore, despite the discovery of new neuroprotective substances, their effectiveness is still not sufficient, due to their chemical constraints like poor solubility or stability and pharmacological limitations leading to drug elimination, peripheral toxicity as well as numerous side effects (Alyautdin et al. 2014). Encapsulation process seems to be a promising strategy overcoming these limitations and increasing the therapeutic efficacy of neuroprotectants (Stockwell et al. 2014). Typically, nanocapsules (NCs) consist of colloidal core surrounded by a polymeric shell that may be further modified, including PEGylation, which prevents nanocapsules from opsonisation and prolongs their half-life (Jokerst et al. 2011; Łukasiewicz et al. 2015; Łukasiewicz and Szczepanowicz 2014; Owens and Peppas 2006). The advantage of PEGylation in the drug delivery to the brain refers to phagocytosis by microglia and nanocarriers’ recognition by other brain immune cells. Since nanocapsules exhibit similarity in their size and morphology to the naturally occurring carriers and their diameters are between 10 and 200 nm, their interaction with cells in the brain is very likely.

A growing body of evidence has pointed out that the active form of vitamin D3—1,25-dihydroxyvitamin D3 (calcitriol), besides the involvement in regulation of the calcium and phosphorus homeostasis in the peripheral tissue, has a potent influence on the brain function. Calcitriol acts via specific nuclear receptors (vitamin D receptor; VDR), which are present in various brain regions as hippocampus, cortex, amygdala, or thalamus (Eyles et al. 2013; Regulska et al. 2007). VDRs may heterodimerize with retinoic acid receptors and act as ligand-activated transcription factors regulating target gene expression in brain (Harant et al. 2000). Among them, genes involved in proliferation, cellular growth, neuronal development as well as neurotransmitter synthesis (DeLuca et al. 2013) have been described. 1,25-dihydroxyvitamin D3 has been shown to regulate neurotrophic signaling such as neurotrophins 3, 4, nerve growth factor (NGF), and glial-derived neurotrophic factor (GDNF), which are important in the survival and migration of developing neurons in the brain. Hippocampal cultures treated with 1,25-dihydroxyvitamin D3 display increased neurite outgrowth (Kesby et al. 2011; Marini et al. 2010). Moreover, some data have underlined that 1,25-dihydroxyvitamin D3 is a potent brain immunoregulator and calcitriol cotreatment with progesterone reduces the brain inflammation after traumatic brain injury (Cekic et al. 2009; Kesby et al. 2011). This ability of 1,25-dihydroxyvitamin D3 may be linked to strongly postulated neuroprotective properties of the secosteroid. Some data have shown that 1,25-dihydroxyvitamin D3 protects neurons against glutamate, 6-hydroxydopamine, and NMDA in neuronal cortical (Taniura et al. 2006) or hippocampal (Brewer et al. 2001) cultures. Moreover, also in in vivo studies the administration of vitamin D3 or its metabolites reduces neurological injury and neurotoxicity in animal systems (DeLuca et al. 2013). The fact that the mechanism of calcitriol action is connected with downregulation of L-type calcium channel expression, stimulation of acetylcholine synthesis and upregulation of intracellular glutathione content suggest that 1,25-dihydroxyvitamin D3 may be useful in the neurodegenerative disorder therapy.

On the other hand, the main drawback of 1,25-dihydroxyvitamin D3 as a potential neuroprotective drug is its ability to induce hypercalcemia and hyperphosphatemia, which in turn may lead to renal insufficiency. Therefore, in previous studies, we tested some synthetic vitamin D3 analogs with low calcemic activity (Regulska et al. 2007; Tetich et al. 2005). We demonstrated that PRI-2191, a low calcemic analog of 1,25-dihydroxyvitamin D3, inhibited staurosporine-induced apoptosis in human neuroblastoma SH-SY5Y cells as well as diminished hydrogen peroxide-, N-methyl-p-aspartate-, and kainate-induced damage.

Alternatively, considering that the neuroprotective effects of 1,25-dihydroxyvitamin D3 do not depend on peripheral calcium homeostasis, it is plausible that some new research strategy overcoming the influence of vitamin D3 on the peripheral calcium homeostasis based on the nanoencapsulation of vitamin D3 should be tested. So far, only a few data deal with the microencapsulation of cholecalciferol as a drug model or as a nutrient supplementation and food fortification. To this end, Luca et al. (2003) tested cholecalciferol-loaded cellulose acetate microspheres as an antioxidizing agent. Moreover, the formulation of calcitriol-loaded microparticles using poly(vinyl neodecanoate-crosslinked-ethyleneglycol dimethacrylate) as a polymer to ensure extended anticancer concentration after local hepatic injection has been demonstrated (Nguyen et al. 2007). Recently, nanoencapsulation of calcitriol was also applied in chemotherapy and as a drug supplier to tumors and metastasized sites (Almouazen et al. 2013; Bonor et al. 2012; Ramalho et al. 2015). Interestingly, cholecalciferol was also encapsulated for oral treatment (Sun et al. 2012).

So far, there are no data concerning the application of calcitriol-loaded nanoparticles as a potential protective agent against the bacterial endotoxin (lipopolysaccharide, LPS)-induced damage in the brain. LPS is a useful tool in studying involvement of inflammatory processes in pathomechanism of neurodegenerative disorders. In this work, we tested calcitriol-loaded nanoparticles with different polyelectrolyte coatings: PLL (Poly(l-lysine hydrobromide)), PLL/PGA (Poly(l-glutamic acid)), and AOT/PLL/PGA-g-PEG (Poly(l-glutamic acid) grafted with polyethylene glycol), in organotypic hippocampal slices as a potential calcitriol delivery system to the brain. The usefulness of hippocampal slices in basic research has been increasing in recent years. The advantage of this technique is the ability to replicate many aspects of the in vivo context. Slices largely preserve the tissue architecture of the brain regions that they originate from and maintain neuronal activities with intact functional local synaptic circulation. In organotypic cultures interactions of multiple cell types in the brain, neurons, astrocytes, and microglia are present (Cho et al. 2007).

Therefore, the aim of the present study was to develop a new formulations of 1,25-dihydroxyvitamin D3 for neuroprotective therapy and to compare their protective potency with that of free 1,25-dihydroxyvitamin D3 in lipopolysaccharide-treated hippocampal organotypic cultures.

## Materials and Methods

Nanocapsules’ constituents: AOT—docusate sodium salt (D4422), PLL—Poly(l-lysine hydrobromide) (P2636), PGA—Poly(l-glutamic acid) sodium salt (P4886), and Me-PEG-NH_2_—methoxypolyethylene glycol amine (6676) were purchased from Sigma-Aldrich. PEGylated polyelectrolyte (PGA-g-PEG) was synthesized according to Szczepanowicz et al. (2010a). Chloroform was purchased from POCH (Gliwice, Poland). 1, 25-dihydroxyvitamin D3 (vit. D3, calcitriol) was synthesized in the Pharmaceutical Research Institute (Warszawa, Poland). All chemicals were used without further purification. Ultrapure water was obtained using Direct Q UV (Millipore).

For organotypic hippocampal cultures, we used Dulbecco’s modified Eagle medium (DMEM), DMEM F-12, horse serum, Hank’s Balanced Salt Solution (HBSS), supplement B-27, N-2, amphotericin B, and Phosphate-Buffered Saline (PBS) from Life Technologies. The Cytotoxicity Detection Kit was from Roche Diagnostic. 3-[4,5-dimethylthiazol-2-yl]-2,5-diphenyltetrazolium bromide (MTT), dimethyl sulfoxide (DMSO), propidium iodide (PI), glucose, penicillin/streptomycin mixture, N-1-naphthylethylenediamine dihydrochloride, sulfanilamide, phosphoric acid, and lipopolysaccharide (LPS, *Escherichia coli*, serotype 0111:B4) were from Sigma-Aldrich.

### Nanocapsules’ Synthesis

**Fig. 1:**
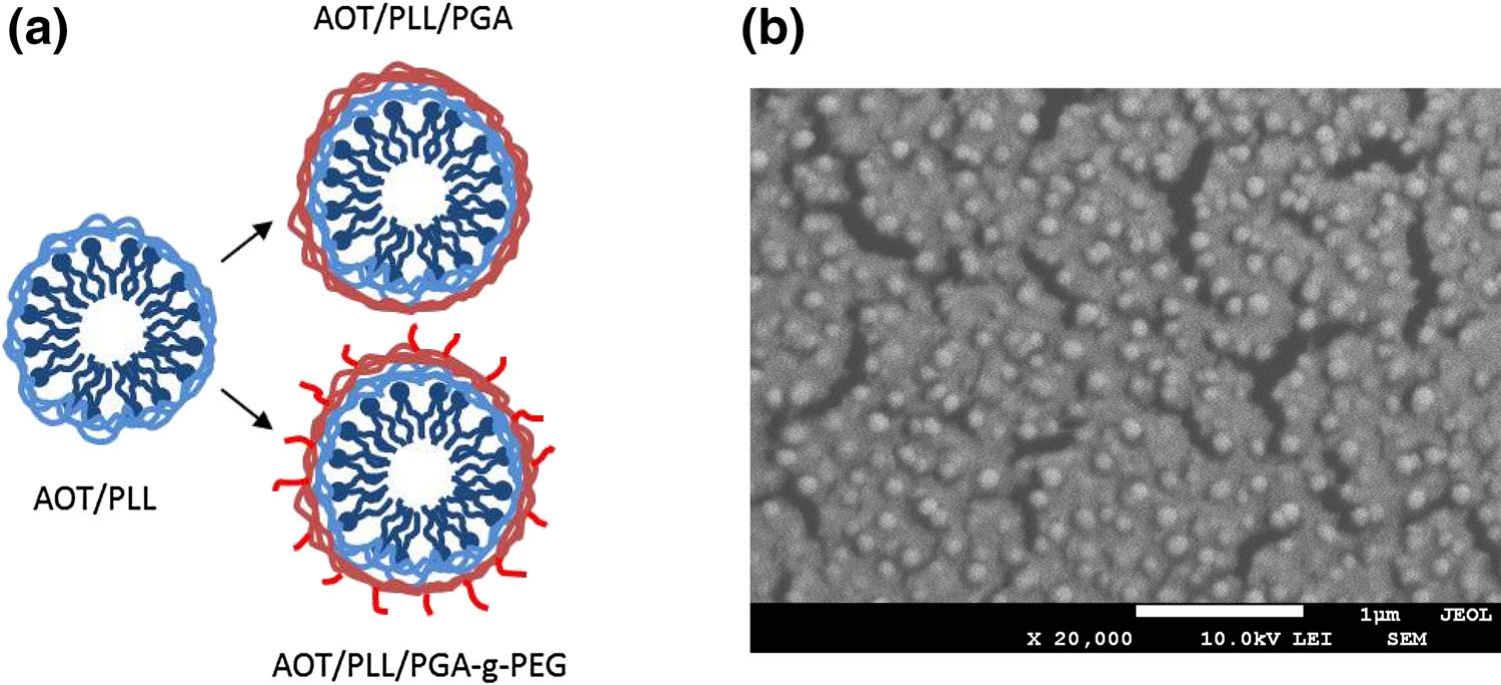
**a** Scheme of nanocapsules with different polyelectrolyte coatings: PLL, PLL/PGA, and PLL/PGA-g-PEG. **b** A cryo-SEM micrograph of AOT/PLL/PGA nanocapsules

Nanocapsules were synthesized as described previously (Karabasz et al. 2014; Łukasiewicz et al. 2016; Piotrowski et al. 2013, 2015; Szczepanowicz et al. 2010b, 2014, 2015). The hydrophobic phase for nanocapsules’ core preparation was formulated by the dissolution of surface-active agent AOT in chloroform (330 mg/ml). Nanoemulsion droplets were formed by adding a fixed 0.025 ml volume of the hydrophobic phase to 50 ml volumes of 0.015 M NaCl aqueous PLL solutions in a wide range of concentration. Next, the proper polycation concentration was determined by the simultaneous zeta potential measurements. It was considered as the most favorable when the zeta potential reached the constant value close to the value of the free polyelectrolyte in the solution. In those conditions, the amount of nonadsorbed polyelectrolyte that can form complexes at the surface of a core was minimized, as most of it was adsorbed, inducing surface overcharging. Subsequently, nanocapsules’ cores were encapsulated in polyelectrolyte shell. Successive layer of the anionic PGA was adsorbed on the positively charged cores using similar saturation procedure. Fixed volume of AOT/PLL cores (8 ml) was added to the polyanion solution in a wide range of concentrations. At a certain PGA concentration, the zeta potential reached the constant value, and this condition was considered as the most appropriate for the nanocapsules’ shell synthesis. Analogously as in the core preparation, recharging of the surface with increasing addition of polyelectrolyte was observed. In the next set of experiments to create PEGylated shell, positively charged AOT/PLL cores were coated with a layer of PGA-g-PEG using the same procedure, by adding PLL terminated cores into filtered PGA-g-PEG solution. Such three types of prepared nanocapsules will hereafter be referred in this work as AOT/PLL, AOT/PLL/PGA, and AOT/PLL/PGA-g-PEG (Fig. 1a).

Importantly, vit. D3-loaded nanocapsules were synthesized as described above except that prior to the emulsification process, calcitriol was dissolved in the AOT-in-chloroform solution (0.25 mg/ml). This concentration corresponds to the 0.3 μM concentration of the calcitriol in the nanoformulation after the synthesis process. Because of the exceptionally low aqueous solubility of vit. D3, 100 % efficiency of encapsulation was assumed. Chloroform was evaporated from suspensions of nanocapsules shortly after synthesis. The amount of the chloroform after evaporation was determined as not exceeding permissible concentration in pharmaceutical products (0.06 µg/ml).

### Nanocapsules’ Size Analysis

The nanoparticle size distribution (hydrodynamic diameter) measurements were performed using Dynamic Light Scattering (Zetasizer Nano Series, Malvern Instruments). Each value was obtained as an average from three runs with at least 10 measurements. All measurements were performed at 25 °C in 0.015 M NaCl.

### Nanocapsules’ Zeta Potential Determination

The zeta potential measurements were performed at 25 °C using Laser Doppler Electrophoresis (Zetasizer Nano Series, Malvern Instruments). Average values were obtained from three runs (with at least 20 measurements) of the instrument. All measurements were performed in 0.015 M NaCl.

### Nanocapsules’ Concentration Measurements

The nanoparticle concentration measurements were performed at 25 °C using Nanoparticle Tracking Analysis (NanoSight NS500, Malvern Instruments).

### Nanocapsules’ Stability Studies

The stability of all types of nanocapsules was evaluated by the periodic measurements of their size (hydrodynamic diameter) and the zeta potential as described above. Each type of nanocapsule suspension was stored at room temperature.

### Nanocapsules’ Visualization

Nanocapsules were visualized using Scanning Electron Microscopy (JEOL JSM-7600F equipped with a cold stage for cryogenic measurements). Samples were prepared by high-pressure freezing.

### Hippocampal Organotypic Cultures (OHCs)

Hippocampal slices were prepared from 6- to 7-day-old Sprague–Dawley rats according to the method of Stoppini et al. (1991) with slight modifications (Kawalec et al. 2011; Kurek et al. 2016). Pups were decapitated and their brains were aseptically and quickly removed to an ice-cold working buffer (96 % HBSS, 3.5 % glucose, 0.5 % penicillin/streptomycin). Next, hippocampi were separated, transferred to Teflon disks, and cut into 400 µm slices using a McIlwain tissue chopper. Afterwards, slices were transposed to Millicell-CM (Millipore) membranes for further growth. Millicell-CM membranes in 6-well plates were preequilibrated with 1 ml of culture medium (50 % DMEM; pH 7,4; 25 % HBSS; 25 % horse serum; 5 mg/ml glucose; 1 % amphotericin B; 0.4 % penicillin–streptomycin; 2 % B-27 supplement). Cultures were initiated in a regular 25 % horse serum-containing medium, which was then gradually (from DIV 4th until 7th) changed to a serum-free medium (50 % DMEM F-12; pH 7.4; 25 % HBSS; 5 mg/ml glucose; 1 % amphotericin B; penicillin–streptomycin; 2 % B-27 and 2 % N-2 supplements). Hippocampal cultures were maintained for 7 days under standard conditions in an incubator (37 °C) with adjustable CO_2_ flow (5 %) before the treatment.

### Treatment of Hippocampal Organotypic Cultures

Hippocampal slices were pretreated for 30 min with various concentrations of free (nonencapsulated) active 1,25-dihydroxyvitamin D3 (vit. D3, calcitriol) or three different types of calcitriol-loaded nanocapsules: AOT/PLL, AOT/PLL/PGA, and AOT/PLL/PGA-g-PEG. Moreover, each type of empty nanocapsules was used. Next, the cultures were stimulated with lipopolysaccharide (LPS; 1 μg/ml). Control slices were treated with vehicle (PBS buffer).

### Determination of Lactate Dehydrogenase Activity

24 h after culture treatment, lactate dehydrogenase (LDH) activity was measured in culture medium using a colorimetric method (Cytotoxicity Detection Kit, Roche Diagnostic GmbH, Germany). The LDH activity was determined by coupled enzymatic reactions. In this test, the amount of formazan salt, formed after the conversion of lactate to pyruvate and then by reduction of tetrazolium salt, is proportional to the LDH activity in the sample. The intensity of the red color formed in the assay, measured at a wavelength of 490 nm, is proportional to LDH activity and also to the number of damaged cells. The data were normalized to the activity of LDH released from control, vehicle-treated slices (100 %) and are expressed as a percentage of the control ± SEM (standard error of the mean).

### Determination of the Metabolic Activity

The metabolic activity of hippocampal slices was determined by the MTT (Sigma-Aldrich, Germany) assay. At 24 h after the treatment, MTT (at 0.15 mg/ml) was added to each well and incubated for 1 h at 37 °C. Next, culture medium was discarded, and 0.1 M HCl in isopropanol was added to dissolve the formazan dye. The absorbance value was measured using a multiwell spectrophotometer (Multiscan, Thermo Labsystem, Finland) at 570 nm. The data were normalized to the absorbance in the control, vehicle-treated slices (100 %) and are expressed as a percentage of the control slices ± SEM (Leskiewicz et al. 2013; Piotrowski et al. 2013; Slusarczyk et al. 2016).

### Flow Cytometry to Detect Cell Death

To confirm the data obtained from the biochemical studies (MTT and LDH tests), 24 h after treatment we stained the hippocampal organotypic cultures with propidium iodide (PI). PI does not cross the cell membrane but stains the DNA released from cells whose cell membrane was disintegrated. Hippocampal slices were transferred to 1.5 ml centrifuge tubes containing 500 μl of ice-cold HBSS. After 5 min of centrifugation (800 rpm), OHCs were incubated with 500 μl of prewarmed Collagenase A solution (30 min at 37 °C), centrifuged, and incubated again with 500 μl of prewarmed 0.05 % Trypsin (20 min at 37 °C). Next, the slices were stained with PI solution (100 ng/ml in PBS) for 5 min at room temperature. Approximately 1 × 10^4^ cells were analyzed using the BD FACS Canto II System and BD FACS Diva™v5.0.1 Software (BD Biosciences, USA) in the fluorescence channel for PE (phycoerythrin, red fluorescence). The PI-negative cells were considered to be undamaged = alive, while the PI-positive cells were considered to be dead. The data were normalized to the results from the control, vehicle-treated slices (100 %) and are expressed as a percentage of the control ± SEM.

### Visualization of Death Cells by Propidium Iodide Uptake

Thirty minutes before the final detection, cultures were supplemented with propidium iodide at a concentration of 10 µM, in order to visualize dead cells. Cytotoxicity (PI staining of cells with damaged membranes) for hippocampal slices in all cultures was detected by fluorescence microscopy (AxionCam MRm, ZEISS, Germany). PI fluorescence reflects staining of necrotic or end-stage apoptotic cells. PI has a maximum excitation wavelength of 536 nm, and the emission of PI in the visual range is 620 nm (Kawalec et al. 2011).

### NO Release Assay

NO secreted in culture medium was measured by a Griess reaction. After 24 h of treatment of hippocampal slices, 50 µl of supernatant from each well was collected and mixed with an equal volume of Griess reagent (0.1 % N-1-naphthylethylenediamine dihydrochloride and 1 % sulfanilamide in 5 % phosphoric acid) in a 96-well plate and incubated for 10 min at room temperature. Absorbance was measured at 540 nm in a microplate reader (Multiscan, Thermo Labsystem, Finland). The data were normalized to the NO released from control, vehicle-treated slices (100 %) and are expressed as a percentage of the control ± SEM.

### Statistical Analysis

Statistical analysis was performed using Statistica 10.0 Software (Statsoft, Tulsa, OK, USA). All data were obtained from three to five independent experiments. All groups were compared by one-way or two-way analysis of variance (ANOVA), followed by Duncan’s post hoc tests for multiple pair-wise comparisons, as appropriate. *P* values less than or equal to 0.05 were considered statistically significant.

## Results and Discussion

### Characterization of Nanocapsules

**Fig. 2:**
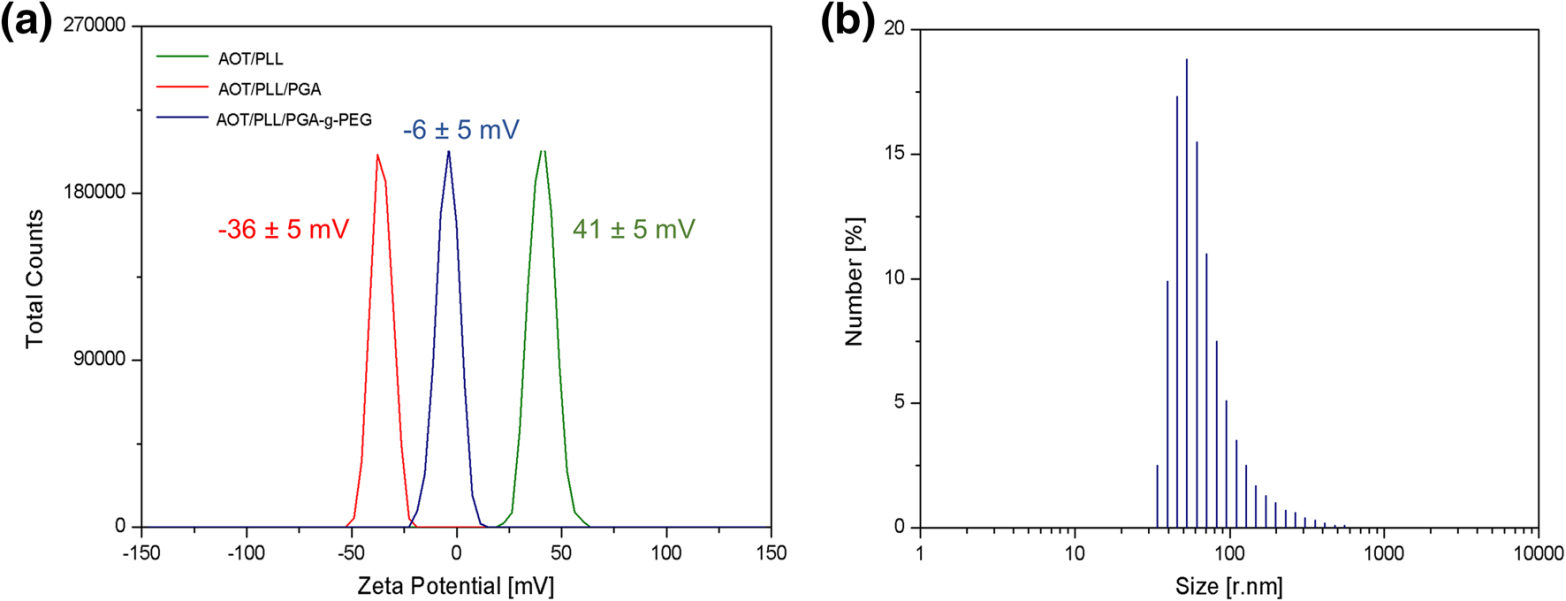
**a** Example of zeta potential distribution of AOT/PLL, AOT/PLL/PGA, and AOT/PLL/PGA-g-PEG nanocapsules. **b** Size distribution of AOT/PLL/PGA nanocapsules by DLS

In our study, the absolute values of the zeta potential of synthesized nanocapsules (AOT/PLL and AOT/PLL/PGA) were higher than 30 mV. Due to the electrostatic interactions, such droplets repel each other and hence exhibit no tendency to aggregation. The zeta potential of nanocapsules with PGA-g-PEG external layer was ~−6 mV. However, steric stabilization mechanisms resulting from adsorbed PEG tails prevent them from aggregation even in a high salt concentration. An example of the zeta potential distribution of AOT/PLL, AOT/PLL/PGA, and AOT/PLL/PGA-g-PEG nanocapsules is shown in Fig. 2a.

The average hydrodynamic diameter of synthesized nanocapsules measured by Dynamic Light Scattering was around 80 nm (Fig. 2b). Particle concentration in the final suspension of nanocapsules, determined by Nanoparticle Tracking Analysis, was approximately 5 × 10^11^ particles/ml. Diameters of most of the synthesized particles observed under Scanning Electron Microscopy were below 100 nm, which is in good agreement with the values obtained by DLS. Some aggregation was observed in the cryo-SEM micrographs (Fig. 1b) due to the freezing of the nanocapsule suspension. Stability of nanocapsules was periodically investigated by measuring the zeta potential and the hydrodynamic diameter in 0.015 M NaCl solution, and no significant changes of these parameters were observed up to 90 days.

### Determination of the Neuroprotective Ability of the Free 1,25-dihydroxyvitamin D3 in Hippocampal Organotypic Cultures

In order to confirm the neuroprotective potential of 1,25-dihydroxyvitamin D3, we examined the effect of this compound in hippocampal organotypic cultures. Hippocampal slice model offers unique advantages over other in vitro platforms. They maintain many aspects of in vivo biology of the examined structure, including functional synaptic connections with preserved organ architecture. Organotypic cultures allow us to conduct a pharmacological manipulation, becoming a very useful method to study molecular pathways or mechanisms underlying several brain disorders (Daviaud et al. 2013; Diekmann et al. 1994; Cho et al. 2004). In addition, hippocampal organotypic cultures seem to be perfect technique to study protective or cytotoxic effect of substances, as they offer the presence of all types of central nervous system’s cells (Cho et al. 2007). Therefore, we selected them for our comparative study regarding the protective effect of free calcitriol and calcitriol-loaded nanoparticles.

In the first set of experiments, we tested the effect of free vitamin D3 in control as well as in lipopolysaccharide (LPS)-treated hippocampal slices. LPS is a primary component of endotoxin from Gram-negative bacteria cell walls (Kettenmann et al. 2011). It binds mostly to toll-like receptor 4 (TLR4), a transmembrane receptor, subsequently inducing intracellular signaling resulting in the activation of mitogen-activated protein kinases (MAPKs) and NF-κB signaling (Rousseau et al. 2013). These factors have been described as the key regulators of proinflammatory cytokine production during inflammation induced by LPS (Zhao et al. 2013). Moreover, LPS induces the nitric oxide production, which plays a role in host defense. However, if NO production gets out of control, damage of host cells occurs due to its cytotoxic potential. Therefore, recently the role of NO as an important regulator in neurodegenerative processes is discussed (Kiemer et al. 2002).

**Fig. 3:**
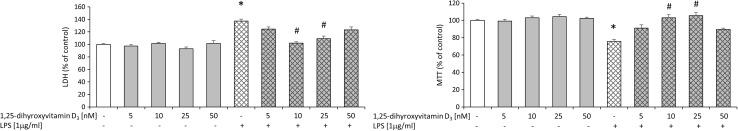
Effect of free 1,25-dihydroxyvitamin D3 and lipopolysaccharide (LPS) on LDH release and MTT reduction in hippocampal organotypic slices. Results are shown as the percent of control ± SEM and the significance of differences between the means was evaluated by the Duncan’s test following a two-way analysis of variance. ^*^ *p* < 0.05 versus control, ^#^ *p* < 0.05 versus LPS

**Fig. 4:**
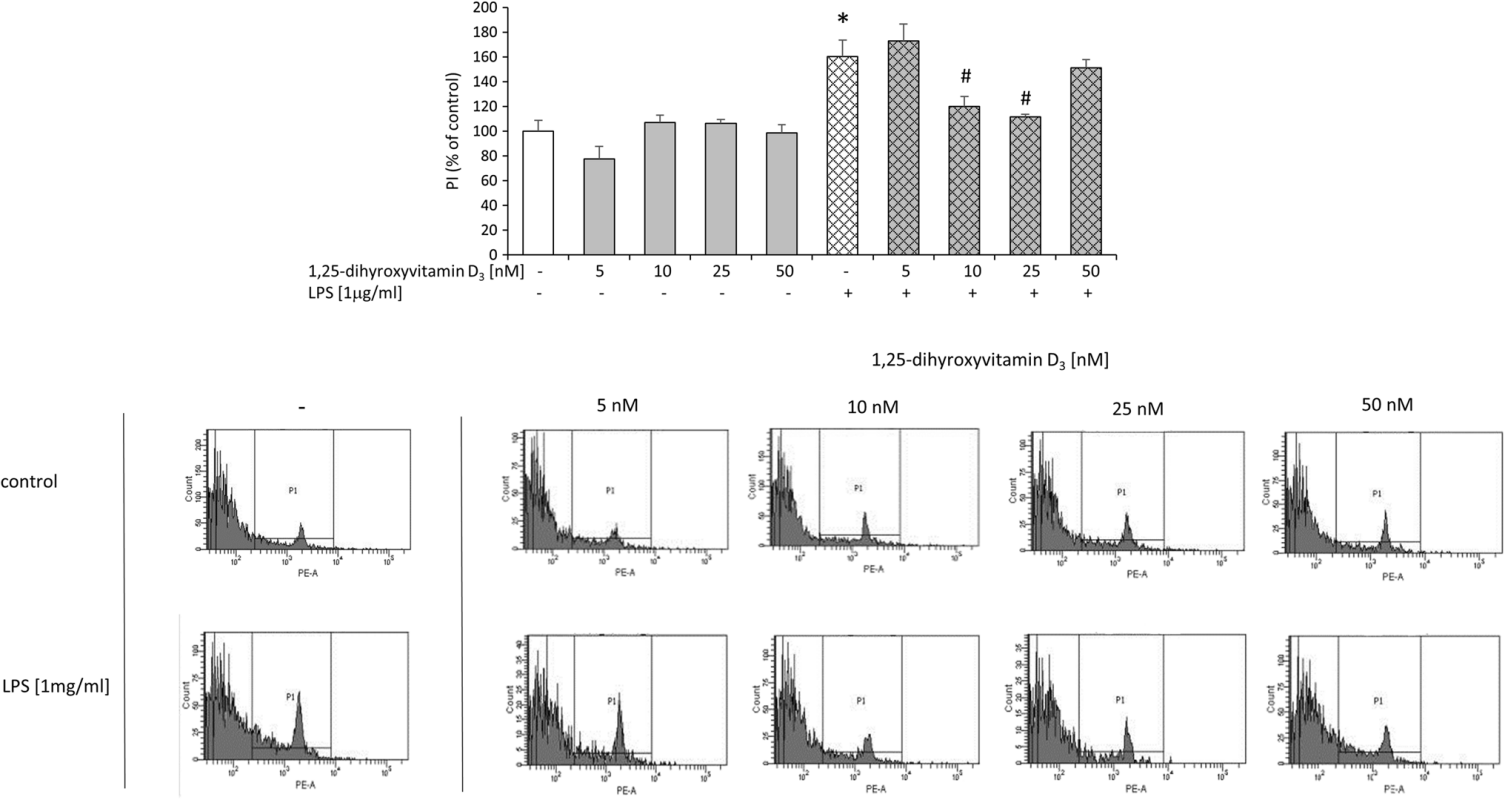
Effect of free 1,25-dihydroxyvitamin D3 on lipopolysaccharide (LPS)-induced damage of hippocampal organotypic slices demonstrated by propidium iodide (PI) staining in flow cytometry method. Results are shown as the percent of control ± SEM and the significance of differences between the means was evaluated by the Duncan’s test following a two-way analysis of variance. ^*^ *p* < 0.05 versus control, ^#^ *p* < 0.05 versus LPS

**Fig. 5:**
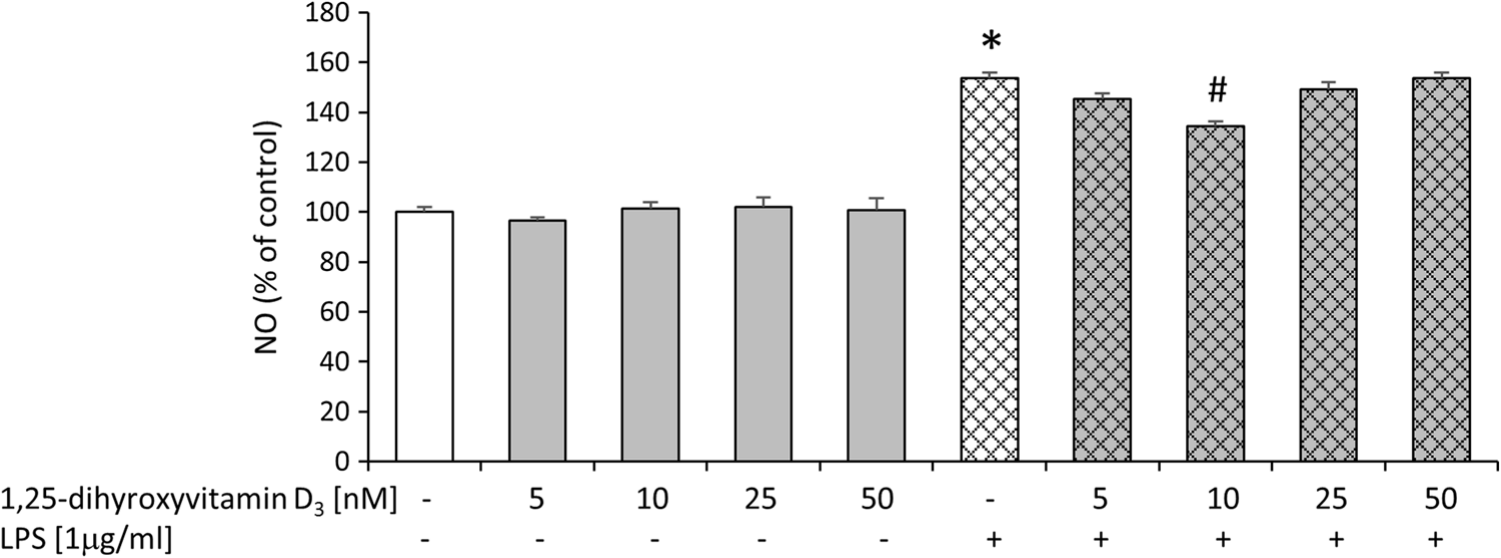
Effect of free 1,25-dihydroxyvitamin D3 and lipopolysaccharide (LPS) on nitric oxide (NO) release in hippocampal organotypic slices. Results are shown as the percent of control ± SEM and the significance of differences between the means was evaluated by the Duncan’s test following a two-way analysis of variance. ^*^ *p* < 0.05 versus control, ^#^ *p* < 0.05 versus LPS

For the examination of potential beneficial ability of active vitamin D3 metabolite, we performed biochemical tests, which evaluated cell death processes (LDH, PI staining), metabolic activity (MTT) as well as nitric oxide (NO) production, 24 h after LPS treatment of hippocampal cultures. Compared to controls, injured cells have impaired membrane integrity, which allow membrane-impermeable molecules to pass through. PI is taken up by damaged cells and intercalated into DNA, whereas increased LDH efflux is detected in injured slices. Therefore, LDH release and PI staining are methods widely used as a quantitative measure of cell death. MTT test is performed to measure the conversion of yellow MTT dye by active mitochondria to form insoluble purple precipitates in live cells. This assay evaluates different aspects of cell death/viability processes than PI uptake or LDH release, because it does not rely on membrane integrity of cells, but rather assesses their metabolic activity. Thus, different tests and finally these multiparameter outcomes may provide an accurate and complete assessment of the vital status of hippocampal organotypic slices (Cho et al. 2007). Initially, we observed that LPS induced cell death processes as revealed by increased lactate dehydrogenase enzyme release (Fig. 3). Bacterial endotoxin also diminished metabolic activity of hippocampal cultures as we found in MTT test (Fig. 3). The damaging effect of LPS in the organotypic slices was further confirmed by enhanced staining with propidium iodide (PI) demonstrated by flow cytometry method as illustrated in Fig. 4. In addition, we visualized the impairment of slices after LPS treatment using PI and observed a substantial amount of (red-colored) death cells. Importantly, incubation with LPS statistically significantly increased nitric oxide (NO) release as reported in Fig. 5. In the next set of experiments, we evaluated the neuroprotective property of active form of vit. D3—calcitriol—using a wide range of its concentrations (5–50 nM). We reported that calcitriol alone did not evoke any significant changes in cell viability as measured by the MTT reduction assay or in cell death as evidenced by both the LDH assay (Fig. 3) and flow cytometry analysis (Fig. 4). Moreover, treatment with 1,25-dihydroxyvitamin in all tested doses had no effect on nitric oxide production (Fig. 5).

Interestingly, pretreatment with calcitriol at the dose of 10 or 25 nM markedly blocked LPS-induced cell death processes as shown in LDH (Fig. 3) and flow cytometry assays (Fig. 4). Moreover, calcitriol (10 and 25 nM), changed, down-regulated by bacterial endotoxin,  metabolic activity in hippocampal slices (Fig. 3). Importantly, only the dose of 10 nM of calcitriol was able to inhibit nitric oxide secretion induced by LPS treatment (Fig. 5). Our findings for the first time demonstrated the dose-dependent neuroprotective properties of active vit. D3 metabolite—calcitriol in hippocampal organotypic cultures. The beneficial influence of calcitriol on the changes caused by bacterial endotoxin should be emphasized.

### Cytotoxicity Tests of Empty Nanocapsules in Hippocampal Organotypic Cultures

Estimation of the neurotoxicity of nanoparticles should be the first step in nanomedical research (Sharma and Sharma 2013; Kabanov and Gendelman 2007). Therefore, we evaluated the cytotoxicity associated with applications of three different types of empty nanocapsules: AOT/PLL, AOT/PLL/PGA, and PEGylated AOT/PLL/PGA-g-PEG. We tested the wide range of each nanoparticles’ dilution—from 5 × 10^11^ particles/ml (1:1), 2.5 × 10^11^ (1:2), 1.25 × 10^11^ (1:4), to 0.63 × 10^11^ particles/ml (1:8) in hippocampal organotypic slices. All assays were done under basal conditions 24 h after treatment.

**Fig. 6:**
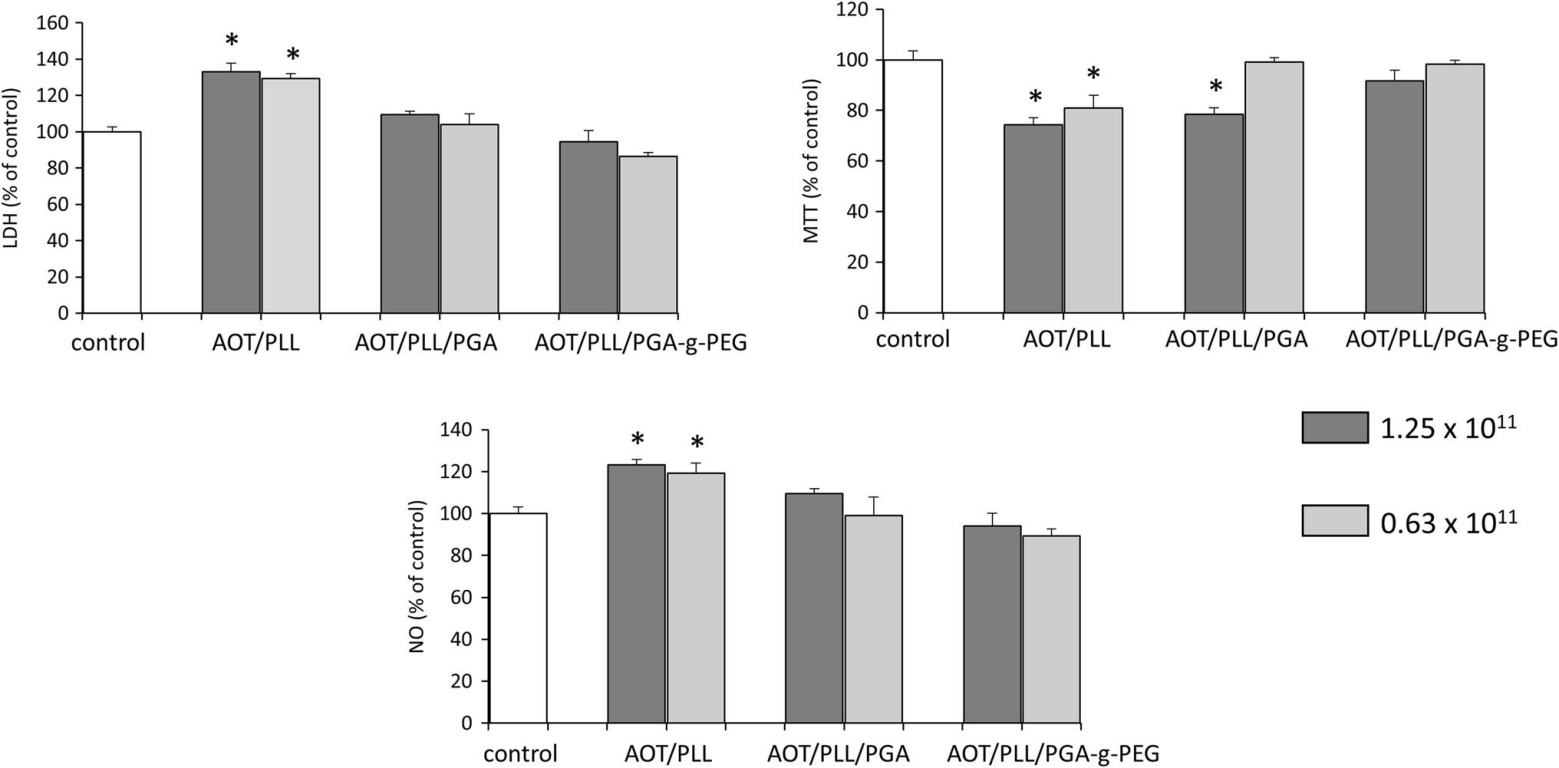
Effect of three different types of empty nanocapsules (AOT/PLL, AOT/PLL/PGA, and AOT/PLL/PGA-g-PEG) in two different concentrations on LDH release, MTT reduction, and nitric oxide (NO) release in hippocampal organotypic slices. Results are shown as the percent of control ± SEM and the significance of differences between the means was evaluated by the Duncan’s test following a one-way analysis of variance. ^*^ *p* < 0.05 versus control

As shown in Fig. 6, AOT/PLL empty nanoparticles were toxic in all dilutions used, including both presented, namely 1.25 × 10^11^ and 0.63 × 10^11^ particles/ml in LDH as well as MTT tests. 24-h treatment with AOT/PLL statistically significantly increased NO production. AOT/PLL/PGA and, AOT/PLL/PGA-g-PEG nanocapsules in the concentration of 5 × 10^11^ as well as 2.5 × 10^11^ particles/ml were toxic in MTT test (data not shown). On the contrary, AOT/PLL/PGA-g-PEG nanocapsules in the concentrations of 1.25 × 10^11^ and 0.63 × 10^11^ particles/ml 24 h after treatment did not affect the lactate dehydrogenase, metabolic activity as well as nitric oxide release in hippocampal organotypic slices. Furthermore, ANOVA test revealed that AOT/PLL/PGA nanocapsules did not affect parameters measured in LDH and NO assays; however, they affected metabolic activity in a concentration-dependent manner. In hippocampal slices at the dilution—1.25 × 10^11^ of AOT/PLL/PGA particles/ml, the metabolic conversion of yellow MTT dye to insoluble purple form was attenuated.

Taking into account the results described above, for the following set of experiments we selected only two types of nanoparticles loaded with calcitriol (AOT/PLL/PGA and AOT/PLL/PGA-g-PEG).

### Determination of the Neuroprotective Ability of Nanocapsules Loaded with 1,25-dihydroxyvitamin D3 in Hippocampal Organotypic Cultures

**Fig. 7:**
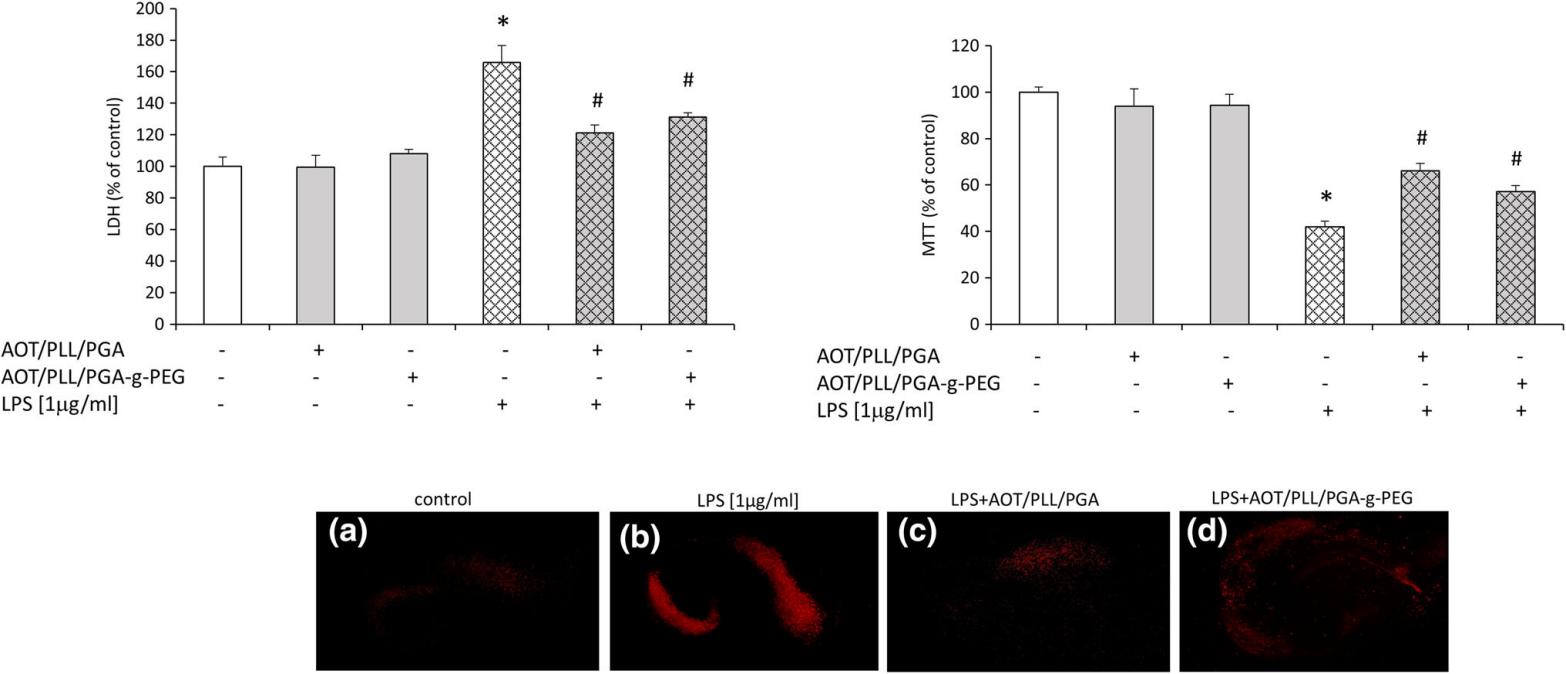
Effect of nanocapsules loaded with 1,25-dihydroxyvitamin D3 (AOT/PLL/PGA and AOT/PLL/PGA-g-PEG) on lipopolysaccharide (LPS)-induced LDH release and MTT reduction in hippocampal organotypic slices. Results are shown as the percent of control ± SEM and the significance of differences between the means was evaluated by the Duncan’s test following a two-way analysis of variance. *p < 0.05 versus control, #*p* < 0.05 versus LPS. Lower panel—pictures A, B, C, D show propidium iodide (PI) staining of hippocampal slices, detected by fluorescence microscopy (AxionCam MRm, ZEISS, Germany)

**Fig. 8:**
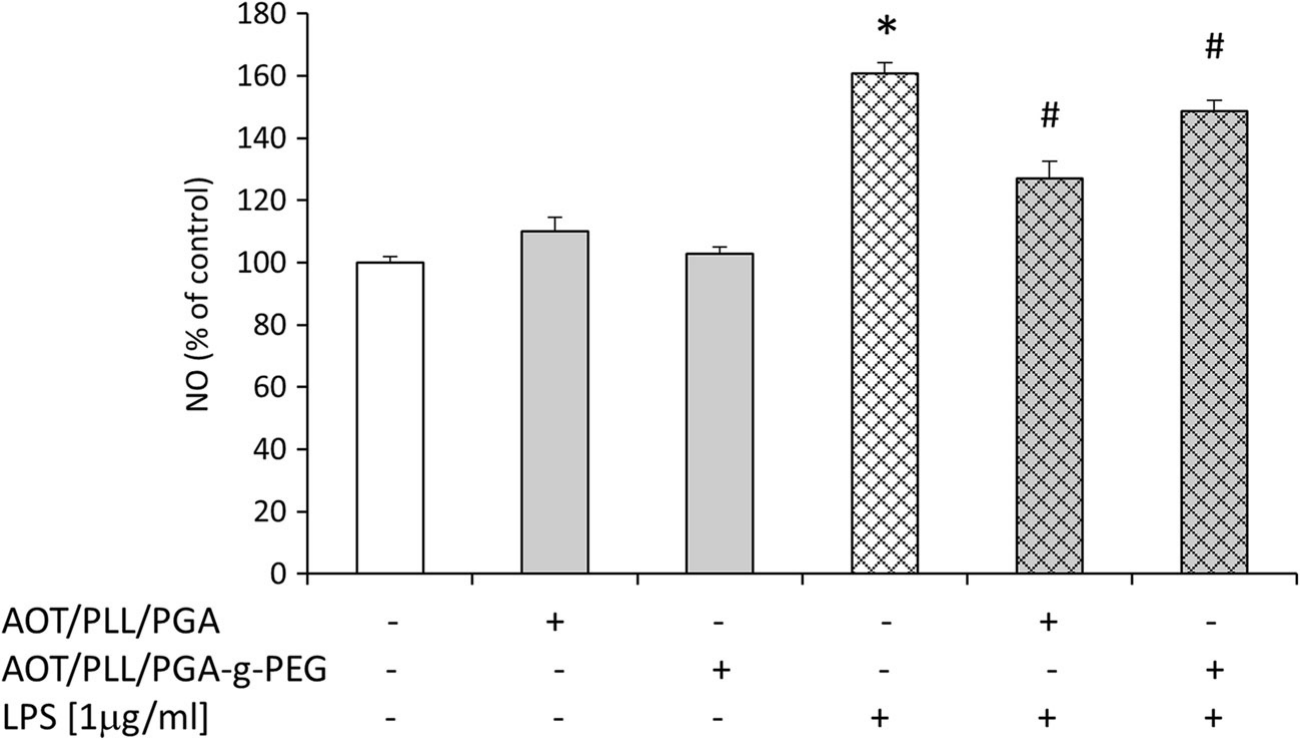
Effect of nanocapsules loaded with 1,25-dihydroxyvitamin D3 (AOT/PLL/PGA and AOT/PLL/PGA-g-PEG) and lipopolysaccharide (LPS) on nitric oxide (NO) release in hippocampal organotypic slices. Results are shown as the percent of control ± SEM and the significance of differences between the means was evaluated by the Duncan’s test following a two-way analysis of variance. ^*^ *p* < 0.05 versus control, ^#^ *p* < 0.05 versus LPS

In this set of experiments, we evaluated the ability of two types of nanoparticles loaded with 1,25-dihydroxyvitamin D3, AOT/PLL/PGA, and AOT/PLL/PGA-g-PEG, to attenuate the damage induced by LPS in hippocampal organotypic slices. The concentration of both types of 1,25-dihydroxyvitamin D3-loaded nanocapsules was 0,63 × 10^11^ particles/ml, which corresponds to the calcitriol concentration of about 3.75 nM. All assays were done after 24 h in the absence or presence of LPS. We observed that in basal conditions (without LPS treatment) neither AOT/PLL/PGA nor AOT/PLL/PGA-g-PEG had any effect on the lactate dehydrogenase release and MTT reduction as shown in Fig. 7. Moreover, the lack of impact of both types of calcitriol-loaded nanoparticles on the NO production in hippocampal slices was demonstrated (Fig. 8).

The main finding of our study is the observation that 1,25-dihydroxyvitamin D3-loaded AOT/PLL/PGA and AOT/PLL/PGA-g-PEG nanoparticles attenuated, upregulated by LPS, release of LDH enzyme, which clearly indicates their protective property in hippocampal slices. The beneficial action of these calcitriol-loaded nanoparticles was confirmed by its ability to normalize the changes in the metabolic activity caused by bacterial endotoxin treatment (Fig. 7). In addition, using PI, we visualized that the impairment of slices after LPS treatment was attenuated by 1,25-dihydroxyvitamin D3-loaded AOT/PLL/PGA and AOT/PLL/PGA-g-PEG nanoparticles (Fig. 7, lower panel). The protective properties of active vitamin D3 metabolite did not depend on the complexes used for nanoparticle stabilization, while both of them statistically significantly suppressed nitric oxide release increased by LPS treatment (Fig. 8). Therefore, it may be postulated that 1,25-dihydroxyvitamin D3 exerts neuroprotective activity, in both free as well encapsulated forms, via inhibition of nitric oxide release. Additionally, it should be strongly underlined that comparing concentrations of the calcitriol free and its nanoform, the loaded nanoparticles show protective effects in the dose of 1,25-dihydroxyvitamin D3 about 3 times lower.

It is worth emphasizing that NO as a pleiotropic molecule plays a role in a number of physiological processes in brain including regulation of proliferation, survival, differentiation of neurons and synaptic activity, neural plasticity, and cognitive functions. However, the pathological effects of NO lead to neuroinflammation and neurodegeneration processes. The NO-associated products resulted mainly from glial activation, which express inducible NOS, produce NO, trigger calcium mobilization from the endoplasmatic reticulum, potentiate the release of vesicular glutamate from astroglial cells, and lead to neuronal death observed in several pathologies like Alzheimer’s, Parkinson’s diseases, multiple sclerosis, or amyotrophic lateral sclerosis (Yuste et al. 2015). Recent data demonstrated the inhibitory effects of vitamin D3 on the NO production in LPS-stimulated BV2 microglial cells. This action of vitamin D3 seems to be related to the suppression of LPS-evoked phosphorylation of p38 kinase through the modulation of vitamin D receptor signaling (Hur et al. 2014). The modulation of the brain NO signaling by vitamin D3 may be a promising target in terms of neuroinflammation and neurodegenerative processes.

## Conclusions

Our study validated the suitability of hippocampal organotypic cultures for evaluating neuroprotective effects of calcitriol and its nanopreparations. This study clearly demonstrated that independently of 1,25-dihydroxyvitamin D3 form, suppression of endotoxin-evoked NO release is involved in its mechanism of action. The higher efficacy of nanoencapsulated 1,25-dihydroxyvitamin D3 than its free form in protecting hippocampal cells against LPS toxicity suggests potential utility of these nanopreparations as a new therapeutic strategy in combating some neurodegenerative diseases, in which inflammation plays an important role.
